# Work as a crime, a resource and a relationship: the confiscation of wages as proceeds of crime

**DOI:** 10.1007/s10611-026-10297-y

**Published:** 2026-07-14

**Authors:** Maayan Niezna

**Affiliations:** School of Law and Social Justice, University of Liverpool, Liverpool, UK

**Keywords:** Criminal law, Labour law, Migration law, Confiscation, Wages

## Abstract

UK law allows the confiscation of wages as proceeds of crime, notably for ‘illegal working’ by undocumented migrants. This article explores the context for criminalising work and confiscating wages, and the arguments against confiscation. It suggests that the confiscation of wages is conceptually, morally, and pragmatically questionable. The justifications for wage confiscation reflect an understanding of work as a finite resource controlled by the state rather than an act or relationship. The arguments against the confiscation of wages reflect the relational and social aspects of work, including the economic necessity to work and work’s potential for coercion and exploitation on the one hand, and dignity and self-realisation on the other. The article further suggests that only when the performance of the job itself is harmful to others may wages be confiscated.

## Introduction

Between 2003 and 2007, Didier Paulet, an undocumented migrant from Côte d’Ivoire, worked in different jobs, including as a forklift truck driver. In 2007, he was arrested and convicted of deception for falsely claiming the right to work in the UK, possessing a false identity document, and driving while unqualified. Alongside a 17-month prison sentence, the prosecution sought to confiscate his ‘benefits’, namely the wages he had earned (CPS [Durham] v N, 2009; Paulet v UK, 2014). The Court of Appeal upheld the confiscation, and Paulet appealed to the European Court of Human Rights, arguing that the confiscation violated his right to property. The Court criticised procedural shortcomings, but did not address the substantive issues of the previous decision, suggesting that the confiscation of wages earned by undocumented workers might still comply with the European Convention on Human Rights (ECHR) (Paulet v UK, 2014).

Confiscation orders may be issued against convicted individuals ‘to recover the financial benefit that the offender has obtained from his criminal conduct’ (Explanatory Notes to the Proceeds of Crime Act, 2002; R v Waya, 2012). By preventing profit from crime, the confiscation regime reflects the moral principle that ‘crime should not pay’. It also serves policy goals such as deterring offenders, strengthening law enforcement, and promoting cost-effective interventions (R v Waya, 2012; Bullock, 2014). According to the Proceeds of Crime Act 2002 (Art. 76[4]), ‘A person benefits from conduct if he obtains property as a result of or in connection with the conduct’. Legally speaking, the confiscation of workers’ wages as proceeds of crime might therefore be justified. If the law prohibits certain work, and someone gains wages from that work, those wages result directly from the offence and would not be paid but for the criminal act. Formally, they are therefore comparable to proceeds from selling drugs or stolen goods. However, in this article, I argue that this approach is conceptually flawed, morally wrong, and inconsistent with human rights and labour rights principles.

The criminalisation of work and the confiscation of wages are underexplored in academic scholarship. This article contributes to their understanding by addressing two important questions: First, what conceptual understanding of ‘work’ underpins its criminalisation and the confiscation of wages; second, what moral, legal, and practical problems arise from such policies. I argue that wage confiscation is generally illegitimate, even when a causal link exists between the wages and a criminal offence. This article therefore contributes to the study of the intersection of work, mobility, and crime, bringing together two theoretical frameworks: the established ‘criminology of mobility’, and the emerging ‘criminology of work’, explored in this Special Issue. The ‘criminology of mobility’ (or ‘border criminology’) examines the increasing use of criminal justice in migration control and the unique forms of criminalisation and penalties applied to migrants (Aliverti, 2015; Bosworth, 2017; Pickering et al, 2018). Confiscation of wages likewise reflects the punitive exclusion of the migrants and the poor (Aliverti, 2015). While citizens may face the confiscation of their wages in some circumstances (for instance, if they commit CV fraud or lie about professional experience in job applications, see R v Andrewes, 2022), the confiscation of undocumented migrants’ wages causes particular harm. Adopted as part of broader migration control, confiscation follows convictions for offences specific to migrants, such as illegal work, or those they are more likely to commit due to their illegal status, like using false documents.

Unlike more common forms of immigration control, such as detention and deportation, wage confiscation has been underexplored within the criminology of mobility. The reasons for this might be the distinctive use of financial rather than physical sanctions, or insufficient theoretical understanding of the nature of work and its relationship with mobility and citizenship. To develop this theoretical understanding, insights from the intersection of criminology of mobility and work are helpful. In this article, I suggest that the criminalisation of work and confiscation of wages reflect an understanding of work as a resource. I then explain the problems with this understanding, arguing instead that labour should be understood as a relationship, combining unfreedom and agency. In this respect, the article contributes to the understanding of the complex relationship between ‘control over mobility and control over labour’ (see Anderson, 2024).

This article is structured as follows. First, it provides an overview of the UK context and ‘hostile environment’ policies. Second, it examines arguments against treating wages as proceeds of crime, showing how punishing those dependent on wage labour is practically ineffective and risks destitution and violation of dignity. Third, it considers the social value and harm of work linked to criminal offences. Finally, it concludes by arguing that wages should only be confiscated when the work itself is harmful, a condition not met in most situations of work by undocumented migrant workers. This article draws on primary legal and policy sources, including legislation, case law, parliamentary debates, and political speeches. Relevant cases were identified through legal database searches for all matters involving wage confiscation, including those unrelated to migrant workers. Parliamentary discussions were analysed to identify and code references to the rationale and understanding of work and wages. By examining the criminalisation of work and the confiscation of wages, the article advances a conceptual understanding of work and wages, and the intersection between the criminology of mobility and the criminology of work.

## The UK context

The offence of illegal working was introduced as part of the UK’s migration control measures known as the ‘hostile environment’. This set of policies, first introduced by the Conservative–Liberal Democrat government of 2010–2015 and continued under the subsequent Conservative government, aimed to make the UK less attractive to undocumented migrants by discouraging illegal entry and overstaying, and by facilitating their removal (Griffiths & Yeo, 2021; Cameron, 2015). These policies denied undocumented migrants access to work, housing, healthcare, and welfare protections. Rather than controlling migration at the territorial border, these measures are taken inside the country, as part of the UK’s ‘everyday borders’ (Anderson, 2013; Lewis et al., 2017; Mayblin et al., 2020; Zedner, 2022). They outsource border control functions and ‘deputise’ third parties—such as employers, landlords, and healthcare practitioners—to enforce migration controls (Griffiths & Yeo, 2021; Zedner, 2022).

In 2015, the Prime Minister promised that illegal working would become a criminal offence and wages would be confiscated as proceeds of crime (Cameron, 2015). The Immigration Act 2016 amended the Immigration Act 1971 to make this promise a reality. The restrictive migration policy that employs economic destitution as one of its migration control measures has, however, much older roots and was promoted by Conservative and Labour governments alike (Aliverti, 2015; Hodkinson et al., 2021).

The criminalisation of work and confiscation of wages, while closely linked, are distinct legal rules. The criminalisation of work denies access to the labour market subject to some conditions, and the violation of the prohibition is punishable under criminal law. According to s24B of the Immigration Act 1971, a person commits the offence of illegal working when they work despite knowing or having a reasonable cause to believe their immigration status does not allow them to work. This may be because they were not granted leave to enter or remain in the UK, if their leave to remain is invalid or no longer effective, or if it does not allow work of a certain kind. The penalty for illegal working is a fine or up to 51 weeks imprisonment in England and Wales, or up to six months in Scotland and Northern Ireland. Additionally, the state may confiscate the wages paid to a person working illegally as proceeds of crime. The ability to seize wages was part of the rationale for introducing the offence of illegal working (House of Commons, 2015a).

Between January and September 2023, the Home Office conducted 4,721 operational visits to enforce the prohibition on illegal working (Independent Chief Inspector of Borders and Immigration, 2024). The year between October 2024 and September 2025 saw a significant increase in raids and arrests targeting illegal working, and the deportation of more than 1,000 migrants (Home Office, 2025b). The former Home Secretary justified an operation targeting the illegal working of delivery drivers on the basis that ‘illegal working damages our communities, cheats honest workers out of employment and defrauds the public purse’ (Burrell, 2023). Enforcement of the prohibition of working is much more common, and the confiscation of wages is rare. Yet, as the case of Paulet and others discussed here show, this is a concerning practice, and understanding its underlying rationale is important. The criminalisation of work and confiscation of wages fit the general use of enforced destitution as a migration control measure adopted in the UK (Hodkinson et al., 2021), and is worsened by the ‘no recourse to public funds’ (NRPF) policy, denying people access to welfare benefits and pushing them further to economic destitution (Qureshi et al., 2020). At the same time, there is limited evidence that the ‘hostile environment’ measures achieve their migration control aims (Griffiths & Yeo, 2021). The negative impact of the confiscation of wages and the criminalisation of work, however, is clear—as the next sections demonstrate.

## Rejecting the confiscation of wages: from work as a resource to work as a relationship

In the parliamentary debate on what would become the Immigration Act 2016 and its provisions criminalising work and enabling the confiscation of wages, politicians justified the exclusion of migrants from the UK’s labour market as preserving employment as a resource for the local population entitled to it. It was suggested, for example, that ‘illegal labour not only exploits the workers whom it employs, but denies work to UK citizens and drives down wages’ (e.g. House of Commons 2015a, col 227, emphasis added), or that ‘the focus of our attention should be on employers who exploit undocumented labour to the detriment not just of undocumented workers, but resident workers who are competing for jobs’ (House of Commons 2015a, col. 219). The former Home Secretary’s statement, referring to anyone who engages in illegal working as one who ‘cheats honest workers out of employment’ (Burrell, 2023), is another example of this approach: honest workers are the rightful beneficiaries of employment, while those working illegally ‘cheat’ to obtain it.

Similar expressions of work as a resource could be identified in legal proceedings concerning the confiscation of undocumented migrants’ wages. In the case of Paulet (2014), the UK government’s argument that ‘persons who had applied to enter the United Kingdom through the visa system would be aggrieved that those who had skipped the queue could retain the savings earned through illegal employment’ (para. 55). The Court further remarked that there was a ‘wider public interest’ in penalising Paulet, as he ‘was deliberately circumventing the prohibition against him seeking remunerative employment in this country in any capacity’ (para. 60). This again echoes the notion that work is a limited resource that should be given to those deserving it: lawful residents and those respecting the rule of law and orderly queues.

Another case concerned a confiscation order against a gangmaster who supplied farms and companies with the labour of undocumented migrants and one of his workers (R v Carter, 2006). The Court considered the provision of labour performed by undocumented migrants and the use of false documents to obtain work. It stated that ‘illegal labour operates so as to prevent those who are entitled to work from being so employed… The proper protection of the right to work and the regulation of the labour market, which operates so as to protect that right to work, provide for an important social objective’ (para. 29). The operation considered in the judgment was determined by the Court to impact both the local community and the migration control measures implemented to ‘supervise asylum seekers’. These statements clearly reflect an understanding of employment as a resource to be justly reserved for citizens.

To conceptualise work as a crime, work is seen as a valued and limited resource controlled by the state and by employers, and obtained by workers. The state and employers control work, which is seen as belonging to them. This ‘belonging’ captures primarily the ability to control and distribute work, but also a broader sense of entitlement over work. This understanding is necessary for criminalising employment obtained following CV fraud or working without a permit. Employment is seen as a resource belonging to the employer in the former case and the state in the latter. They are entitled to divide it as they see fit, and those obtaining it unfairly are committing a public wrong. The best way to understand the statements above, concerning cheating, queue jumping, and denying work from those who deserve it, is if work is understood as a resource. This perspective reflects what some economists refer to as the ‘lump of labour’ fallacy, which assumes there is a fixed amount of work available (e.g. Ruhs & Vargas-Silva, 2014; Wels & Macnicol, 2019), and therefore treats work as a scarce resource to be managed and distributed by the state. However, if work is understood not as a resource, but as a relationship or an act, such notions of cheating or jumping the queue make little sense. For instance, no one would suggest there is not enough family (relationship) to go around, or that runners deny running or painters deny painting (act) to other people entitled to it. To consider work as an exclusive entitlement of citizens because there is not enough work to go around is to consider work as a resource.

Work as a resource owed by the state to its citizens can also be identified in human rights law, although the scope of the right to work is not entirely clear, and it is subject to some criticism (Mundlak, 2007; Collins, 2015; O’Cinneide, 2015). Article 6(2) of the International Covenant on Economic, Social and Cultural Rights (ICESCR, 1966) requires states to adopt relevant policies, develop vocational training and implement other techniques to enable ‘full and productive employment under conditions safeguarding fundamental political and economic freedoms to the individual’ (United Nations, 1966, Art 6(1)). The literature on the right to work considers a right to a job – as in, a right not to be unemployed – and a correlative duty of the state to provide work (Collins, 2015; O’Cinneide, 2015). This would be a positive right to work, as opposed to the negative right to work requiring the state not to prevent one from working.

In R v Carter (2006), the Court of Appeal rejected the claim that, as far as confiscation orders are concerned, wages were a special category. This position makes sense if work is a resource, a position that I challenge here. However, as I will show, because work cannot be reduced to a resource, and because wages are unique, their confiscation as proceeds of crime should be rejected for conceptual, moral and policy reasons. To understand the conceptual problem in the confiscation of wages (and to some extent, in the criminalisation of work), the idea of ‘work as a resource’, demonstrated above in statements of politicians and courts, should be replaced with a more accurate understanding: ‘work as a relationship’.

The focus on work as a resource distributed by the state reflects a failure to conceptualise the nature of work fully and correctly. It maintains a fiction of a conceptual separation between the worker and labour, as noted by Marx’s (1867/1990) emphasis that the worker’s labour power ‘exists only in his living body’ (p. 272). Polanyi (2001) further explains that: ‘Labor is only another name for a human activity which goes with life itself, which in its turn is not produced for sale but for entirely different reasons, nor can that activity be detached from the rest of life, be stored or mobilized’ (p. 75). Work therefore does not exist independently of the worker’s self. It is, first and foremost, an activity performed by the worker. This basic understanding of work is overlooked in the conception of work as a resource underlying the criminalisation of work and confiscation of wages.

Conceptually, work should be understood not as a ‘thing’ the worker obtains, but as an act performed and, more broadly, as a relationship. The nature of this relationship explains why criminalising work in general and confiscating wages in particular are conceptually, morally, and pragmatically wrong. Recognising the relational nature of work is conceptually important for accurately understanding work and acknowledging the humanity of the parties involved. Practically, understanding work as a relationship highlights power relations between employers and workers (or capital and labour), justifying labour law interventions like collective action and statutory protections. The relational aspects of work reflect broader social structures and economic relationships. These include employers’ authority and control over workers and the hierarchical structures enforcing discipline and compliance, but also norms concerning cooperation, trust, and confidence.

The relational nature of work is inherent in any account of labour rights or potential claims by workers, including for instance individual or collective claims concerning wages, excessively long working hours, or sexual harassment in the workplace. None of these claims could be understood without recognition of the power relations between the parties and, therefore, the relational nature of work. Some rights concerning access to work (such as discrimination in hiring) might be conceptualised as concerning work as a resource. However, even access and discrimination cases may have a relational aspect. When access to work is subject to union membership (‘closed shop’ – see e.g. Davies, 2009), it too reflects the nature of work as a relationship: relationships between workers, between workers and job-seekers, and between unions and employers. Work as a relationship also explains its role in the relationship between the state and citizens, for whom work is perceived as a duty, part of their contribution to society, and their integration in it (Weeks, 2011; Anderson, 2024).

## Work as a relationship of limited freedom

While work enables varying degrees of agency, it is not entirely freely chosen. Even under ideal conditions, work may involve significant unfreedom: in entering the work relation, in keeping it, and in terminating it. In particular, one reason why wages are unique is that people have little choice but to work in order to afford their basic needs. The confiscation regime assumes criminals make rational calculations, weighing risks before committing crimes. If crime profits are confiscated, the incentive to commit crimes disappears. While this assumption is questionable in general (Bullock, 2014), it is particularly problematic when the ‘crime’ is working without a permit or when fraud seems necessary to secure employment, such as when hiring criteria are discriminatory. Wage labour, as Marxist and other writers argue, is not truly free but is driven by economic compulsion and a lack of alternative income (Marx & Engels, 1978; Miles, 1987; Zwolinski, 2020; Adams, 2022). Most people must work to meet their basic needs, even if jobs are unsatisfactory. As the means of production are owned by capital, workers have little choice but to sell their labour power to provide for their needs. The result is an inherent power imbalance between employers and workers, which may be reduced or increased by governmental interventions.

Work often involves significant physical and emotional hardship, and may be poorly paid, routine, dirty, dangerous, or difficult (Anderson, 2013; Collins, 2015; Cruz, 2020). While economic necessity exists even in fulfilling jobs, it is especially pronounced in the lower end of the job market, where wages and conditions are poor. This is particularly relevant for migrant workers, who are often confined to dirty, dangerous, and difficult jobs offering low pay and poor conditions that local workers reject (Piore, 1979; Anderson, 2013). Migrants’ vulnerability to exploitation in this context is well-documented, and arises from factors such as language barriers, unfamiliarity with local laws and customs, discrimination, unrecognised skills, and limited support networks (Anderson, 2010, 2013; Bloch & McKay, 2016; Boucher, 2023). The vulnerability of both documented and undocumented workers is also shaped by laws and policies restricting labour market access, legal aid, and labour protections (Lewis et al., 2017; Shamir, 2017; Broad & Gadd, 2023; Mantouvalou, 2023). For these workers, the confiscation of wages is unfair and ineffective as it ignores the unfreedom in the constrained choice to enter work relations.

Recognising work as a result of economic necessity challenges the moral and policy justifications for wage confiscation. While working without a permit may be a deliberate strategy for some migrant workers, it is a choice made within constraints (Ruhs & Anderson, 2010). For those working illegally, constrained agency often stems from a lack of legal avenues to earn a living or meet basic needs (Ruhs & Anderson, 2010; Lewis et al., 2017; Mayblin et al., 2020). This applies to workers fleeing unemployment in their home countries or seeking work abroad when local opportunities cannot provide a dignified living or cover essential costs such as healthcare and education. Although these circumstances may not meet the legal threshold of duress, criminalising and punishing such actions should still be rejected. Acknowledging economic necessity as central to the nature of work should lead to greater caution in confiscating wages, particularly when a worker’s situation is desperate. This is especially relevant for migrants in low-wage sectors.

### Criminalisation increases the risk of exploitation

The economic necessity to work also raises important issues concerning the risk of exploitation. When the offence of illegal working was discussed in Parliament, MPs from various parties suggested that the offence would increase vulnerability, drive people into exploitation and deter migrant workers from reporting exploitation. They argued that the fear of imprisonment would become a threat to be used by gangmasters and employers. Some noted that the threat of sanctions was likely to deter even workers who are not committing an offence, as they might be unaware of their rights and status.

These concerns also reflected the tension between the Immigration Bill and the Modern Slavery Act, passed only a few months earlier and celebrated as a major step to tackle severe exploitation. Several MPs expressed concern about the application of the Immigration Bill to victims of modern slavery (House of Commons, 2015a), or pointed to the contradiction between the objectives of the Modern Slavery Act and the offence of illegal working (House of Commons 2015b; see also Davies, 2016; Fudge, 2018; Hodkinson et al., 2021). Concerns over the risk of exploitation were also prominent in the advocacy efforts of non-governmental organisations (NGOs) before the passing of the Act and in scholarship and the opinions of expert bodies since (e.g. Davies, 2016; Fudge, 2018; GRETA, 2021; Boucher, 2023). NGOs have since reported cases of exploited immigrants afraid of complaining, and the deportation of potential victims of exploitation (LEAG and FLEX, 2020).

The government rejected the concerns that criminalising work would increase vulnerability. Government representatives claimed that tackling the illegal labour market would reduce the pull factors leading migrants into exploitative work and reduce exploitation (UK Parliament, 2015; House of Commons, 2015a). In the second reading of the Bill in the House of Lords, the Minister of State at the Home Office argued that ‘illegal workers and the victims of exploitation are not necessarily the same people. Illegal workers, in most cases, have come to the UK for personal economic gain, circumventing our existing immigration laws… Where there are victims, the system is loaded with safeguards’ (House of Lords, 2015 [col 2451–2452]). No clear evidence was offered to support this claim, which did not fit even the government’s own framing of the Bill and its objectives. The Home Secretary herself suggested the Bill was meant to tackle both illegal working and exploitation, and that those tempted to come to the UK illegally might later ‘find themselves living and working in dangerous and degrading conditions’ (House of Commons, 2015a [col 197]).

Criminalised workers are often undocumented migrants in precarious labour sectors, many of whom already face extreme exploitation. Victims of exploitation frequently do not identify as such or report their situation to the authorities (Van Meeteren & Hiah, 2019; Cockbain & Tompson, 2024). If they do, contacting the authorities to report abuse may expose them to threats of detention, deportation, and loss of wages (Hodkinson et al., 2021; Broad & Gadd, 2023). Criminalisation and confiscation further discourage complaints about exploitative conditions, and provide abusive employers with a further coercive tool to enforce unacceptable working conditions. Evidence from various contexts supports the claim that migrant workers need to be able to complain about violations of their rights without fear of migration control measures (Hastie, 2017; Migrant Justice Institute, 2023). This notion of a safe reporting mechanism or a ‘firewall’ between labour and migration enforcement was proposed by various actors (ECRI, 2016; FLEX, 2019; JCWI, 2023; ILO, 2022), but not adopted in the UK.

In the UK, undocumented workers are also barred from claiming labour rights in courts due to the defence of illegality. Under this rule, courts are prevented from providing a remedy in civil cases when a person’s cause of action is based on an illegal act. While the Supreme Court made an exception in cases amounting to trafficking in persons or those very close to trafficking (Hounga v Allen, 2014), it does not apply to those considered to freely choose to work without a permit (Fudge, 2018; Bogg, 2020). This narrow interpretation ignores the problem explored here, namely, the economic necessity and dependency that lead many to work without a permit.

Overall, most of the parliamentary debates around the criminalisation of work under the Immigration Bill focused on the risk of deterring vulnerable migrants from reporting abuse, and the risk that the law now gives employers another threat to hold over them. The focus on the pragmatic consequences of the Bill might have been a practical, strategic choice, and these objections to the confiscation of wages as ‘proceeds of crime’ are certainly convincing and should be central concerns for policy analysis. Yet, the sole focus on pragmatic considerations might also be the result of a lack of conceptual clarity and moral investigation of the meaning and significance of work itself. The arguments proposed in this article may help address this oversight.

### State sovereignty, criminalisation and confiscation

It may be argued that the restriction on migrants’ right to work results from the right of sovereign states to determine the conditions of entry and stay within their territory. From this perspective, the UK’s confiscation of wages is a legitimate migration control measure aimed at deterring and punishing illegal entry and stay. This was the position of the government when introducing this measure (Cameron 2015; House of Commons, 2015a; House of Lords, 2015). Some may also consider economic sanctions less injurious to migrants than detention and deportation. To this, one could respond in several ways.

First, other sanctions against illegal entry and stay, such as detention and deportation, are already part of the law. The addition of wage confiscation might be disproportionate and amount to double punishment. Second, while deprivation of liberty is usually a more severe sanction than economic sanctions, some migrants might consider confiscation of wages worse than short detention. The fear of sanctions may differ due to personal circumstances, country of origin, and length of stay (Ruhs & Anderson, 2010). Furthermore, different sanctions, such as financial loss, abuse and threat of deportation, may combine to coerce workers into exploitative employment and deter them from reporting abuse (Yea & Chok, 2018). As shown above, this risk was highlighted in the context of the UK offence of illegal working. Third, even if state sovereignty was accepted as a reason to introduce the severe measures of criminalisation and confiscation, there is little evidence to suggest the ‘hostile environment’ measures, including the offence of illegal working, work in practice (Griffiths & Yeo, 2021). The Home Office (2020) itself doubted whether access to the labour market acts as a ‘pull factor’ for asylum seekers. Griffiths and Yeo (2021:530) suggest understanding the Hostile Environment as ‘an ideological stance’ rather than ‘an evidence-based, ends-driven policy approach’, and Aliverti (2015) highlights the populist, performative, and symbolic role of immigration offences that might not reflect the basic justification of criminal law. Adopting harsh means to achieve a public aim is one thing; cruelty for cruelty’s sake is another.

Finally, while the state may be entitled to introduce migration control measures, there are legal limits to this power, key among them is the protection of human rights (CCPR, 1986; Goodwin-Gil, 1989; Juridical Condition and Rights of the Undocumented Migrants, 2003). As we have seen, the criminalisation of work and confiscation of wages create a real risk of slavery and forced labour, themselves severe violations of human rights. Beyond this, however, it also violates the notion of work as a human right and an important source of dignity and social well-being.

## Work as a human right and a source of dignity

While the work relationship is one of inherent unfreedom, the economic necessity that forces one to work as a means of livelihood also makes work an important aspect of dignity. The right to work as a human right was recognised under the Universal Declaration of Human Rights (UDHR). According to Art 23(1), ‘Everyone has the right to work, to free choice of employment, to just and favourable conditions of work and to protection against unemployment’ (United Nations, 1948). Work as a source of livelihood and a means to provide for oneself and one’s family was reflected in Article 23(3) of the UDHR, stating that ‘everyone who works has the right to just and favourable remuneration ensuring for himself and his family an existence worthy of human dignity, and supplemented, if necessary, by other means of social protection’. The International Covenant on Economic, Social and Cultural Rights (ICESCR) likewise developed the right to work and distinguished between the right to work, and related rights or rights at work such as equal pay, remuneration allowing decent living, health and safety, and the right to form and join trade unions (United Nations, 1966). As explained above, the confiscation of wages might violate not just the right to work but also rights *at* work; in particular, by deterring workers from leaving exploitative conditions or demanding respect for rights such as health and safety or decent remuneration.

The right to work under the ICESCR includes the following:

The States Parties to the present Covenant recognize the right to work, which includes the right of everyone to the opportunity to gain his living by work which he freely chooses or accepts, and will take appropriate steps to safeguard this right (Art 6[1]).

It does not include entitlement to a job for which one does not meet the necessary conditions, or for a job anywhere in the world. Indeed, it may be argued that migrants’ right to live in dignity does not require gaining a living specifically in the host state. This point raises an important distinction between a right to work in the migrants’ host state, and their right to wages for work already performed. Refugees’ right to engage in gainful employment is protected under the 1951 Geneva Convention (United Nations, 1951; UNHCR Executive Committee, 1988; Costello & Ó Cinnéide, 2021), but migrant workers usually require a permit to enter the host state and work there. Even the International Convention on the Protection of the Rights of All Migrant Workers and Members of Their Families recognises various limitations to migrants’ right to employment opportunities (United Nations, 1990).

Yet, once migrants perform work, the law requires that their rights at work be protected. Thus, the International Labour Organization (ILO) recognises the rights of migrants without regular status to ‘equality of treatment…in regards to rights arising out of past employment as regards remuneration, social security and other benefits’ (ILO, 1975; see also Davies, 2016). The rights of migrants without regular status and the application of the principle of non- discrimination to their situation were also recognised by the Inter-American Court of Human Rights (Juridical Condition and Rights of the Undocumented Migrants, 2003). Once work was performed, the confiscation of wages denies workers, and possibly their families, the right to live in dignity, to an adequate standard of living, and freedom from inhumane and degrading treatment and destitution.

However, violation of dignity and basic rights results not just from the confiscation of wages, but also from the broader criminalisation of work by undocumented migrants. Its combination with exclusion from access to welfare benefits and social support may lead to economic destitution, as people can neither work nor rely on benefits. Such destitution was evidenced by interviews with undocumented migrants in the UK (Jolly, 2018; Qureshi et al., 2020). In the UK, the House of Lords considered the case of withdrawing support from asylum seekers who were forced to sleep rough and beg for food. In its judgment, the Court found the prohibition on inhuman and degrading treatment (under Article 3 of the ECHR) applied to ‘asylum-seekers who are not only forced to sleep rough but are not allowed to work to earn money and have no access to financial support by the state’ (Limbuela v SSHD, 2005, para. 60). In several other countries, legal proceedings resulted in allowing asylum seekers access to work when the alternative is destitution (Costello & Ó Cinnéide, 2021). The confiscation of wages of those not entitled to social support from the state would result, at least in some cases, in similar destitution and inhuman and degrading treatment. It would therefore violate the rights of migrant workers.

## Impact on broader social relations

The relational nature of work is manifested not just in the relationship between workers and employers or among workers themselves, but also in broader social relations, in personal relations and friendship with colleagues, and in workers’ sense of social contribution through their work. While the confiscation of wages might violate workers’ dignity by pushing them to destitution, the confiscation of wages, or the broader issue of criminalisation of work, violates workers’ dignity in other ways.

Different readings of the meaning of work might link it to finding fulfilment, self-realisation, dignity or independence (Mundlak, 2007; Collins, 2015). Being treated as a commodity would not promote one’s self-realisation, and should not be considered as a human right. Costello and Ó Cinnéide (2021) note that work is instrumentally valuable, in enabling ‘individuals to participate in social, economic, and political life, and crucially to earn a livelihood’ (p. 954), and intrinsically valuable to a sense of dignity and self-worth. Scholarship on the meaning of work, including from philosophy, psychology and sociology, suggests that the contribution of work to life is not only limited to salary, but functions also as a ‘human need’ (Yeoman, 2014; see also Elster, 1986). Thus, Gheaus and Herzog (2016) identify four ‘goods of work’ in addition to wages. The first good is attaining ‘various types of excellence’, including knowledge, technological achievements and the development and exercise of skills—or, as Veltman (2016) describes, ‘capabilities’. The second good is ‘social contribution’, which could be made through paid or voluntary work (Gheaus & Herzog, 2016; see also Veltman, 2016). The third good is an experience of community – interacting, collaborating and spending time with other people, and through working with others participating in the community’s social, economic and political life (Gheaus & Herzog, 2016; O’Cinneide, 2015; Penner, 2015). Last, work allows people an opportunity to gain social recognition and grant one a sense of belonging and contribution (Gheaus & Herzog, 2016; Veltman, 2016).

Veltman (2016) also suggests two additional purposes of work. First, the development or exercise of virtues such as ‘honor, dignity, pride, dependability, industriousness, cooperativeness, self-discipline, and self-reliance’ (p. 122). Second, the integration of aspects of one’s life, relationships, identity, and life story, as ‘elements of meaningfulness in life and in work often overlap’ (p.131). These narratives integrate not just the individual life story, but also their social relations and belonging. This last aspect echoes, for example, the common tendency of many people to introduce themselves to new acquaintances by first describing what they do or their job title.

Defining work as a criminal offence risks turning a potentially important aspect of one’s identity into something shameful. The criminalisation of work disrupts its role in fostering belonging and social recognition, instead linking community to criminality or signalling exclusion. Gheaus and Herzog (2016, p. 79) argue that ‘a minimum requirement of justice with regard to social recognition is that one should not have to work in a job that undermines one’s ability to receive social recognition’. Criminalising work removes this social good, replacing it with shame. Even if those working illegally do not see their work as dishonest or immoral, social responses can still erode their sense of dignity.

The position of some MPs in the debates on the Immigration Bill echoed the importance of work in providing for oneself and one’s family and contributing to the host society (House of Commons, 2015a). For instance, Lord Rosser criticised the government’s position and its contradiction of the positive value of hard work in stating that ‘this is one group of working people who will not be lauded by the Government but will instead now be criminalised and removed from the country for the offence of working hard’ (House of Lords 2015, Col 2445). Similarly, Burnham argued that ‘working to put food in your kids’ mouths should never be a criminal offence’ (House of Commons 2015b, col 272), and Crowly that ‘asylum seekers should be allowed to work so that they are able to provide for themselves and their families adequately. We believe they should be able to work so that they are able to make a contribution to the country they call home’ (col 271).

At the same time, some scholars have also criticised the centrality of work, emphasising that self-realisation, fulfilment, meaning, and community can exist outside of work (Weeks, 2011; Mundlak, 2007). Others agree but note that dependence on work for subsistence and the time it consumes leave little room to pursue these goods elsewhere (Yeoman, 2014; Gheaus & Herzog, 2016). Conversely, work provides structures and resources to develop capabilities and find meaning that are harder to access outside of it (Yeoman, 2014). Given its economic necessity and prominence in most lives, the self-realisation and meaning derived from work cannot be ignored. This might be true even in low-paid, routine, dirty, dangerous, or difficult jobs held by undocumented migrants, or in cases like Andrewes, who committed fraud to secure employment. A worker may have fabricated their CV, yet still find fulfilment in performing their tasks and solving problems. The initial crime need not obscure the value of the work itself.

Like criminalisation, the confiscation of wages as proceeds of crime may also hurt workers’ dignity in important ways. While payment is not inherently essential to self-esteem or self-realisation (unpaid interns or volunteers may derive significant satisfaction from their voluntary work, for instance), dignity, self-esteem, or self-realisation is often closely tied to paid work. For many, including those doing a job that is not particularly interesting or enjoyable, supporting themselves and their family through work reflects their values and gives a sense of respect and self-esteem (Nickel, 1978; Elster, 1986; Menges et al., 2017). For previously exploited workers, in particular, work may restore a sense of pride and self-sufficiency (e.g. Hacker & Cohen, 2012). Research shows that some find meaning, social status, contribution, and even moral duty in criminalised work, such as people smuggling (Achilli, 2018; Sanchez & Zhang, 2020). This may suggest that at least some workers sense self-esteem and dignity from work performed against legal restrictions or obtained by dishonest means, but that otherwise contributes to society.

As Rogers (2013) suggests, wages may have an ‘expressive effect’ and indicate moral equality and social worth. In a society equating monetary value with social or personal worth, wages can validate the value of one’s labour and status (Solow, 1990). In this respect, confiscating wages risks undermining these dimensions of dignity at work, serving as a reminder of worker’s low status or a denial of their social contribution. Moreover, the criminal stigma is attached to undocumented migrant workers not just because of what they do, but also because of who they are: poor migrants who, due to their nationality, are not exempted from visa requirements (Spena, 2014; see also Aliverti, 2015). The CPS (Crown Prosecution Service) policy not to pursue confiscation when a formerly undocumented worker acquired a right to remain in the UK (GRETA 2021) further emphasises that confiscation marks those excluded from social membership.

## Performance, value and harm

Justifying the confiscation of undocumented migrant workers’ wages, the courts referred to a ‘public interest’ in penalising those working without a valid permit (R v Carter, 2006; Paulet EWCA, 2009). Indeed, the courts position in such cases seems harsher than in other cases of confiscation, where consideration of proportionality and performance of the work were given more weight (R v Waya, 2012; R v Andrewes, 2022). The Supreme Court considered cases of migrants working illegally as ‘criminal enterprise cases’, stating that ‘the provision of illegal labour does not constitute restoration of value’ (R v Andrewes 2022, [37]).

I disagree. Although the causal link between fraud and wages is clear, confiscation concerns value, and the value of work performed should not be dismissed. It is hard to imagine, for instance, agricultural labour where a worker’s status affects the value of the crops planted or picked. As the court noted, many employers would not care about their workers’ legal status (R v Andrewes, 2020). The fraud may be punishable for breaching social norms of truth and honesty, but it does not alter the value created by the workers. The same reasoning would apply in other cases. The distinction between the initial misrepresentation (or other offence resulting in access to work) and performing the work itself is also helpful in determining when, if ever, wages should be confiscated. This raises legal, moral, and practical considerations that raise the question of whether the confiscation of wages is ever justified. One might empathise with poor migrants who lack acceptable livelihood alternatives, but what about less sympathetic cases? Should a worker like Andrewes, who committed CV fraud to obtain a high-salaried management job for which he did not qualify, be allowed to keep his salary from the company he defrauded? What about members of criminal organisations making their living trafficking drugs or stolen goods? Or hired killers?

In responding to these questions, I suggest focusing on the social value and harm of the work itself. Many jobs produce social value. Being a forklift driver, farm worker, or manager is not morally wrong; such work is generally beneficial or at least not harmful. The issue lies with the worker’s status or claims when applying, not with job performance. It reflects what Ashworth (2008: 420) defined as ‘performing a legitimate activity in an illegitimate way’. As long as the work itself, regardless of how it was obtained or the worker’s legal status, is not harmful, wages should not be confiscated. However, if performing the work causes harm, confiscation may be justified. This view reflects the recognised role of harm in justifying criminalisation and criminal law enforcement (Mill, 2003; Feinberg, 1987; Duff & Marshall, 2014). Relevant harms include physical, psychological, impact on private or public entities, and direct financial harm (Greenfield & Paoli, 2013; Canning & Tombs, 2021). Profits or wages from selling stolen goods could thus be confiscated, whereas wages from legitimate work by unauthorised workers could not. Some currently legal sectors, such as the arms trade or the tobacco or meat industry, are argued to produce social harm (Canning & Tombs, 2021). In such sectors, wages may not be confiscated as long as the sector is legal.

In the migration context, direct harm related to the work must be distinguished from general indirect harms attributed to irregular migration. The Home Office identified illegal working as a ‘high harm’ category of immigration crime as part of a harm matrix used between 2007 and 2022 (Aliverti, 2012; Home Office, 2025a). Considering the criminalisation of migration, Costello (2020) examined potential types of harm caused by irregular migration. She suggested arguments can refer to harm to the migrants themselves (who are being exploited), harm to local workers whose salaries and conditions are brought down, and violation of the territorial integrity of the state. Harm to the workers themselves is irrelevant to confiscation, and Costello effectively showed why arguments based on these types of harm are unconvincing. However, an important angle is missing from this analysis; namely, the perception of harm due to migrant’s use of employment opportunities that would otherwise be available to the local population. This argument reflects the exact rhetoric of work itself as a finite resource used to justify the confiscation of undocumented migrant workers’ wages. The previous section explained why this potential argument should be rejected.

## Conclusion

When an unscrupulous employer withholds wages from migrant workers, knowing their undocumented status will prevent them from complaining, he is perpetrating criminal exploitation. When the state does the same, it is considered a law enforcement and migration control measure. In both cases, a worker is deprived of their salary. The value they produce and their basic labour rights are denied. The harms the confiscation of wages creates are more severe than the ones it aims to address. Yet, like other serious harms caused by ‘state crimes’, they are overlooked within the standard analysis of criminality (Canning & Tombs, 2021).

This article demonstrated the harms resulting from the confiscation of wages, a topic neglected in academic scholarship. As workers have few alternatives to working (even without a permit), punishing a choice resulting from economic compulsion is morally wrong. It is also likely to discourage migrant workers from reporting exploitation, potentially increasing the risk of severe exploitation, including forced labour and servitude, and leading to further economic destitution. Moreover, the confiscation of wages also carries an expressive effect (compare Rogers, 2013 on minimum wage), denying the basic dignity, moral equality, and social worth of migrants and the work they do. I have therefore argued that, aside from situations where the work itself is harmful, the confiscation of wages should be considered illegitimate.

An important theoretical contribution of this paper is the development of a clearer conceptualisation and understanding of work. The perception of work as a finite resource controlled and distributed by the state and employers explains the rationale for the criminalisation of illegal working and the confiscation of wages from those obtaining work in ways considered illegitimate. Its contrast with the understanding of work as a relationship combining social harm and social value, as explained above, raises important questions for further research: in which other policy areas is work understood as a resource, and in which is it understood as a relationship? How does this understanding impact access to work, industrial relations, and related policies, such as welfare and benefits? Identifying the areas in which work is seen as a resource may also shape responses and interventions addressing policies based on the same erroneous understanding.

## Data Availability

Not applicable.
